# Supplementary material for: Metagenome-wide characterization of shared antimicrobial resistance genes in sympatric people and lemurs in rural Madagascar
Source: PeerJ. 2024 Jul 30;12:e17805. doi: 10.7717/peerj.17805 (PMC11296303; doi:10.7717/peerj.17805)
Supplement: Supplemental Information 2 [file peerj-12-17805-s002.docx]

**Supplemental Analysis**


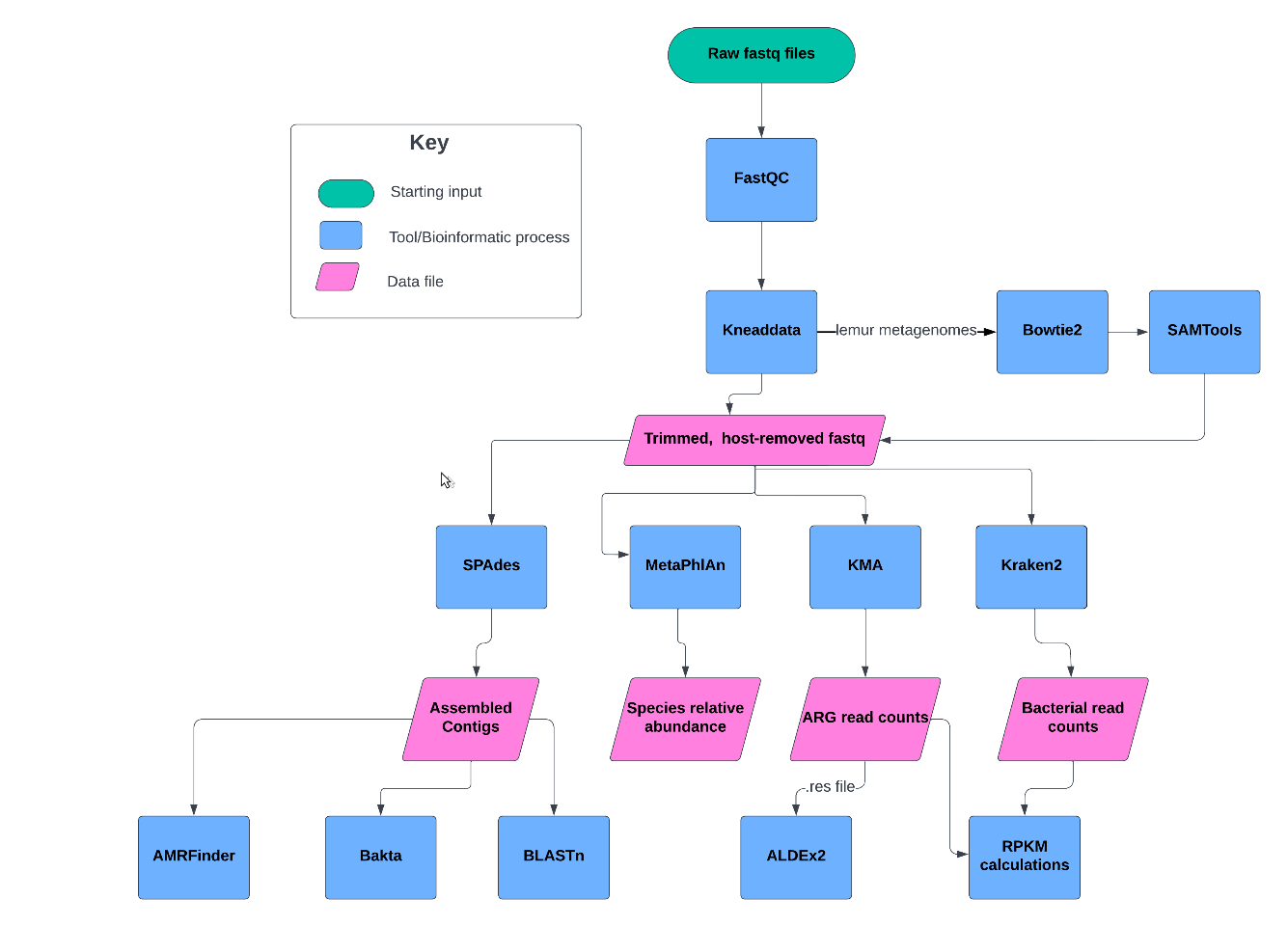


**Figure S1.** A schematic of the overall bioinformatic workflow of sampled human and lemur metagenomes.

***Quality comparison of Kraken2-classified Lemur microbiome metagenome reads***

The nucleotide sequence quality between unclassified and classified reads was compared to understand its impact on categorize metagenomic reads into taxa for lemur sequence. Reads were run through a comprehensive Kraken2 database (PlusPFP release date 2024-01-12) which includes taxa Refseq markers for bacteria, human, virus, plasmids, plants, protozoa, fungi, and UniVec_Core sequences. Kraken2 was run with the flags --unclassified-out to generate unclassified reads from each read pair sequences, --classified-out for the classified reads per paired sequence, --report to generate the quality report, and --paired to indicate the two paired end sequences for each sample. A quality report was produced summarizing the paired-end sequences of each sample.


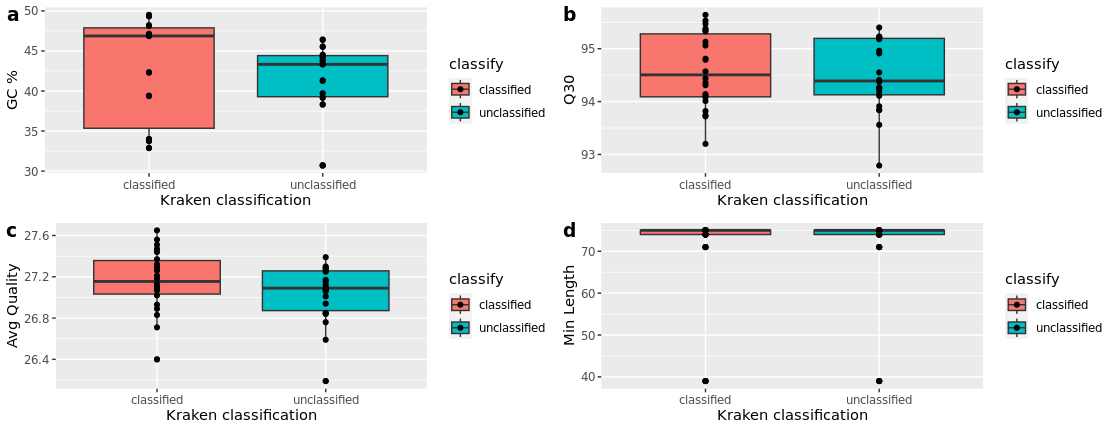


**Figure S2**. Comparison of quality metrics between classified and unclassified reads from lemur microbiomes. Boxplots were generated from the values of each sequence of a read pair per sequence after analysis with Kraken2 against the Kraken PFP database for the following four metrics: (a) The GC percentage per sequence, (b) percentage of reads at Q30 score, (c) the average Phred sequence quality, and (d) the minimum sequence lenght

***Evaluation of differential abundance calculations using ALDEx2 for ARGs grouped by gene families and primary antibiotic class association***

Raw reads of ARGs were assessed with ALDEx2 to identify differential gene abundances between humans and lemurs, accounting for sparseness and composition in the data. Differential abundance grouped at the gene family and the antibiotic class the gene is associated with conferring resistance against. The Aldex2 central log transformation (clr) function was used to generate final analyses. To test the effect of scaling on the outcome of differential abundance, a sensitivity analysis was performed using the function aldex, which introduces different levels of uncertainty using the gamma parameter.


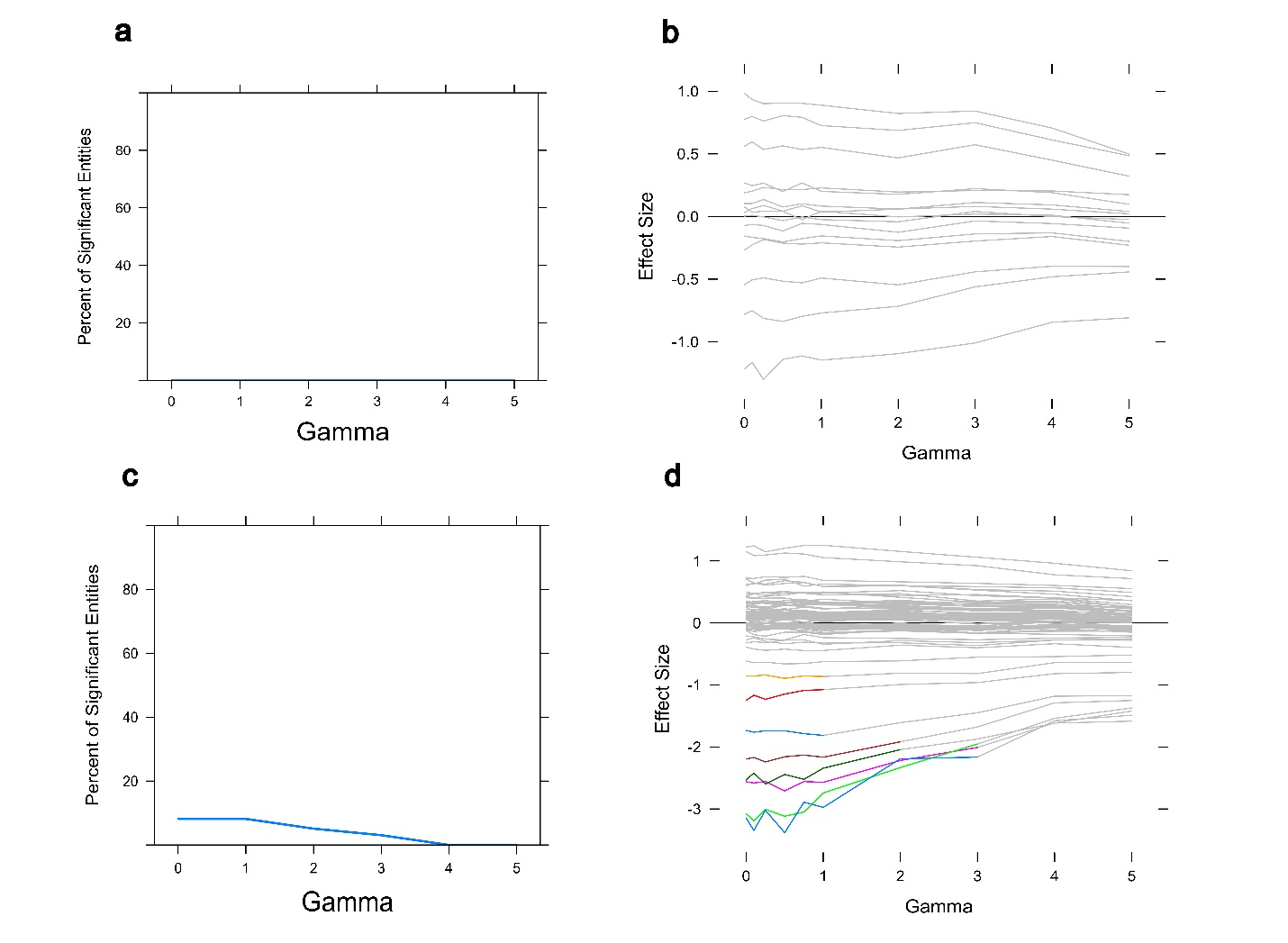


**Figure S3. Sensitivity of detecting significant differential abundances at different levels of uncertainty using ALDEx2**. Raw read abundances of ARGs were summed by either antibiotic class associated with resistance (a-b) or by the gene family associated with the positive allele hit (c-d). Plots were generated using the plotGamma function in ALDEx2 (v.1.35.0). (a) The percent of significant entities for reads grouped by antibiotic class was not significant for any value of gamma. (b) Individual lines indicate unique entities of antibiotic classes and their corresponding effect size of differential abundance at different values of gamma. Gray indicates that an effect size is not significant. (c) The percent of significant entities for reads grouped by gene family was significant between gamma values of 0 and 3. (d) Individual colored segments indicate significant effect sizes for differential expression of eight unique gene families up through a gamma value of 1. Five entities remain significant up through gamma = 2, and two remain positive up through gamma = 3
